# Impact of frequent Bcl-2 expression on better prognosis in renal cell carcinoma patients

**DOI:** 10.1038/sj.bjc.6601454

**Published:** 2004-01-06

**Authors:** T Itoi, K Yamana, V Bilim, K Takahashi, F Tomita

**Affiliations:** 1 Division of Molecular Oncology, Department of Signal Transduction Research, Graduate School of Medical and Dental Sciences, Niigata University, Asahimachi 1-757, Niigata 951-8510, Japan

**Keywords:** Bcl-2, apoptosis, renal cell carcinoma (RCC), prognosis

## Abstract

Previously, we reported that Bcl-2 was frequently expressed in renal cell carcinoma (RCC) specimens, but p53 mutation was a rare event. However, it was unclear whether Bcl-2 positivity was associated with the clinicopathological characteristics and prognosis in RCC. Therefore, we investigated the expression of Bcl-2 protein and its roles in 101 RCC specimens. In addition, the proliferation index (PI), apoptotic index (AI), caspase-3 and p53 expression were examined. The immunohistochemical method was applied for Bcl-2, caspase-3 and p53 protein expression. To investigate the proliferation activity and apoptosis of tumour cells, PI and AI were calculated based on Ki-67 and terminal deoxynucleotidyl transferase-mediated dUTP-biotin nick-end labelling (TUNEL)-positive cells, respectively. Bcl-2 expression was detected in 72 out of 101 (71.3%) specimens. Bcl-2 positivity was inversely correlated with PI (*P*<0.0001) and AI (*P*=0.0074). Furthermore, Bcl-2 positivity was significantly correlated with better survival (*P*=0.0014), and was associated with lower stage (*P*=0.0301) and grade (*P*=0.0020). In RCC, frequent Bcl-2 expression was correlated with favourable character without higher PI and AI. Thus, Bcl-2 expression might be applied as a novel predictor of better prognosis in RCC patients.

Tumour growth depends on two main factors, cell proliferation and cell death by apoptosis (Korsmeyer, 1992). Apoptosis is a form of cell death characterised by morphological, biological and genetic features. Abnormalities of apoptosis may lead to uncontrolled cellular proliferation and ultimately carcinogenesis. Several studies have reported significant correlations between apoptosis and prognosis in malignant tumours including lung cancer (Macluskey *et al*, 2000), breast cancer (Gonzalez-Campora *et al*, 2000) and oesophageal cancer (Shibata and Matsubara, 2001).

Renal cell carcinoma (RCC) has unique clinical characteristics. These include spontaneous metastasis regression after removal of the original tumour, late metastasis after curative surgery and a higher resistance to conventional chemotherapy and irradiation. Thus, it is difficult to predict prognosis based on conventional factors – stage and grade. There is a strong necessity for more precise predicting parameters. Previously, three studies reported the correlation of apoptosis with overall survival of RCC patients (Tannapfel *et al*, 1997; Yoshino *et al*, 2000; Richter *et al*, 2002), but these results were controversial. Tannapfel *et al* (1997) stated that high apoptotic index (AI) was significantly related to poor prognosis and high grade of RCC, but others stated that high AI was significantly related to better prognosis.

The proto-oncogene bcl-2 is implicated in the regulation of cell death by inhibiting apoptosis, while the tumour–suppressor gene p53 and caspases are involved in the induction and execution of apoptosis. Previously, we reported that urothelial transitional cell cancer is characterised by high expression of bcl-2 and mutant p53 (Watanabe *et al*, 1994; Bilim *et al*, 1996, 1998); furthermore, Bcl-2 is frequently expressed in RCC specimens, but p53 mutation is a rare event in RCC (Tomita *et al*, 1996). However, it was unclear whether Bcl-2 positivity was associated with clinicopathological characteristics and prognosis in RCC. Although Bcl-2 expression has also been intensively investigated in other studies, its role in RCC progression and RCC patients prognosis remains controversial (Lipponen *et al*, 1995; Vasavada *et al*, 1998; Huang *et al*, 1999; Sejima and Miyagawa, 1999; Zhang and Takenaka, 2000; Uchida *et al*, 2002). Results of these studies are summarised in Table 1

**Table 1:** Studies with regard to Bcl-2 expression in RCC

**Author (year)**	**Number of patients**	**Bcl-2 positive**	**Bcl-2 positive in clear cell cases**	**Others**
Lipponen *et al* (1995)	104	21/104 (20.2%)	NA^a^	Bcl-2 expression was related to low T category
Tomita *et al* (1996)	25	17/25 (68.0%)	NA	
Vasavada *et al* (1998)	35	33/35 (94.2%)	26/28 (92.9%)	Bcl-2 expression was related to high tumour grade
Huang *et al* (1999)	33	23/33 (69.7%)	NA	
Sejima (1999)	53	34/53 (64.2%)	NA	
Zhang (2000)	70	24/70 (34.3%)	19/56 (33.9%)	
Uchida *et al* (2002)	112	52/112 (42.0%)	NA	

a Not assessed.

. Therefore, we decided to investigate the expression of Bcl-2 protein in RCC and clarify its diagnostic and prognostic significance.

In this study, we investigated the immunohistochemical expression of Bcl-2, caspase-3 and p53 protein directly involved in the regulation of apoptosis, Ki67 marker for proliferation, detected apoptotic cells by TUNEL staining, and evaluated the relation of these factors to clinicopathological characteristics and prognosis in patients with RCC. Although Bcl-2 was shown to be related to decreased survival and malignant potential in urothelial transitional cell cancer (Bilim *et al*, 1996), oesophagus cancer (Hsia *et al*, 2001), prostate cancer (Bauer *et al*, 1996) and other cancers, we demonstrated here that Bcl-2 is an independent favourable prognostic factor in RCC patients.

## MATERIALS AND METHODS

### Patients

A total of 101 patients (69 men and 32 women, mean age 58 years, range 36 – 83) with RCC, who underwent partial or radical nephrectomy between 1991 and 2000 at our institution, were studied (Table 2

**Table 2:** Patients characteristics

Mean age (range) years	58.0 (38–83)
Male/female	69/32
	
*T stage*	
T1	43
T2	19
T3	36
T4	3
	
*Grade*	
1	34
2	58
3	8
Unknown	1
	
*Histological type*	
Clear cell	76
Granular cell	7
Spindle cell	3
Pleomorphic	4
Bellini duct	1
Mixed	10

). The mean follow-up period was 58.7 months (range 1 – 161).

### Immunohistochemistry

Immunohistochemical staining was performed using the avidin – biotin peroxidase complex method, as described previously (Tomita *et al*, 1993). Briefly, 5 *μ*m sections using a cryostat from frozen tissues were placed on poly-L-lysine-coated slides or silane-coated slides, and fixed in cold acetone for 10 min. The sections were incubated for 15 min in 20% normal sheep serum at room temperature, followed by avidin and biotin for 10 min. The sections were then incubated with the primary antibodies for 1 h at room temperature, except for Ki-67, which was applied overnight at 4°C. We used the following primary mouse monoclonal antibodies: anti-human Bcl-2 protein (1/50 dilution, Dako, Glostrup, Denmark), p53 Ab-2 (1/200 dilution, Oncogene Research Products, Boston, MA, USA), Caspase-3 (CPP32)Ab-3 (1/100 dilution, NeoMarkers, CA, USA) and Anti-human Ki-67 antigen (1/100 dilution, Dako). Next, the sections were incubated with biotinylated sheep anti-mouse immunoglobulin (Amersham, Buckinghamshire, England) for 15 min at room temperature. After incubation with streptavidin – horseradish peroxidase (HRP) (Amersham) for 15 min at room temperature, the sections were peroxidase-stained in 0.05% diaminobenzidine tetrahydrochloride (DAB) in 100 ml 50 mM Tris-HCL with 0.01% H_2_O_2_. They were finally counterstained with Meyer's haematoxylin solution (Wako, Japan) and mounted with Mount-Quick (Daido Sngyo Co., Ltd, Japan) after dehydration in graded ethanol and xylene.

The proliferation index (PI) was expressed as a percentage of the Ki-67 positive cells in the tumour cells, with atleast 500 cells counted on the several fields for each section.

The evaluation of staining were assessed by two observers (V Bilim and T Itoi), who were blinded to the clinicopathological characteristics of the patients.

### Detection of apoptosis

Apoptotic cells were detected by TUNEL staining (*In Situ* Apoptosis Detection Kit, TAKARA, Japan), according to the manufacturer's instructions, as described previously (Bilim *et al*, 2000). The AI was expressed as a percentage of the TUNEL-positive cells in the tumour cells, with atleast 500 cells counted in the several fields for each section.

### Immunoblotting

Immunoblotting was performed as described previously (Tomita *et al*, 1996). Briefly, tissue samples were lysed in lysis buffer (0.1 M Tris-HCl pH 8.0, 5 mM EDTA, 0.15 M NaCl, 1% Triton X, 1 mM aprotinin, 1 mM leupeptin and 1 mM PMSF). These lysates were cleared by centrifugation at 15 000 r.p.m. for 30 min at 4°C. A measure of 10 *μ*g of each protein was electrophoretically separated on a 15% SDS – polyacrylamide gel. Immunoblots were blocked with 10% skimmed milk in TBS at room temperature for 1 h followed by the primary mouse monoclonal antibody: anti-human Bcl-2 protein (1/200 dilution, Dako, Glostrup, Denmark). Then, the blots were incubated with biotinylated sheep anti-mouse immunoglobulin, followed by incubation with streptavidin – HRP for 1 h. Biotin–streptavidin–HRP complexes were detected using the ECL Western blotting system (Amersham), according to manufacturer's instructions. To confirm that an equal amount of proteins were applied to each lane, the blots were subjected to staining for *β*-actin (Oncogene Science) as a control.

### Statistical analysis

All statistical analyses were performed using commercially available software (StatView 4.5,. Abacus Concepts, Berkeley, CA, USA). χ^2^-tests were used to assess the correlation between Bcl-2 expression and clinicopathologic parameters. The Mann – Whitney *U*-test was used to analyse the difference in frequencies of AI and PI between clinicopathological parameters, and the relation of Bcl-2 expression with AI or PI. Pearson's correlation was used to compare AI and PI. The survival curves were constructed using a Kaplan – Meier analysis, and the differences between curves were tested using the log-rank test. Cox proportional hazards model was used in univariate and multivariate analysis. The cutoff values of AI and PI correspond to the mean values of each of the indices. Values exceeding these cutoffs were considered high.

## RESULTS

### Expression of Bcl-2, caspase-3 and p53

**Figure 1:**
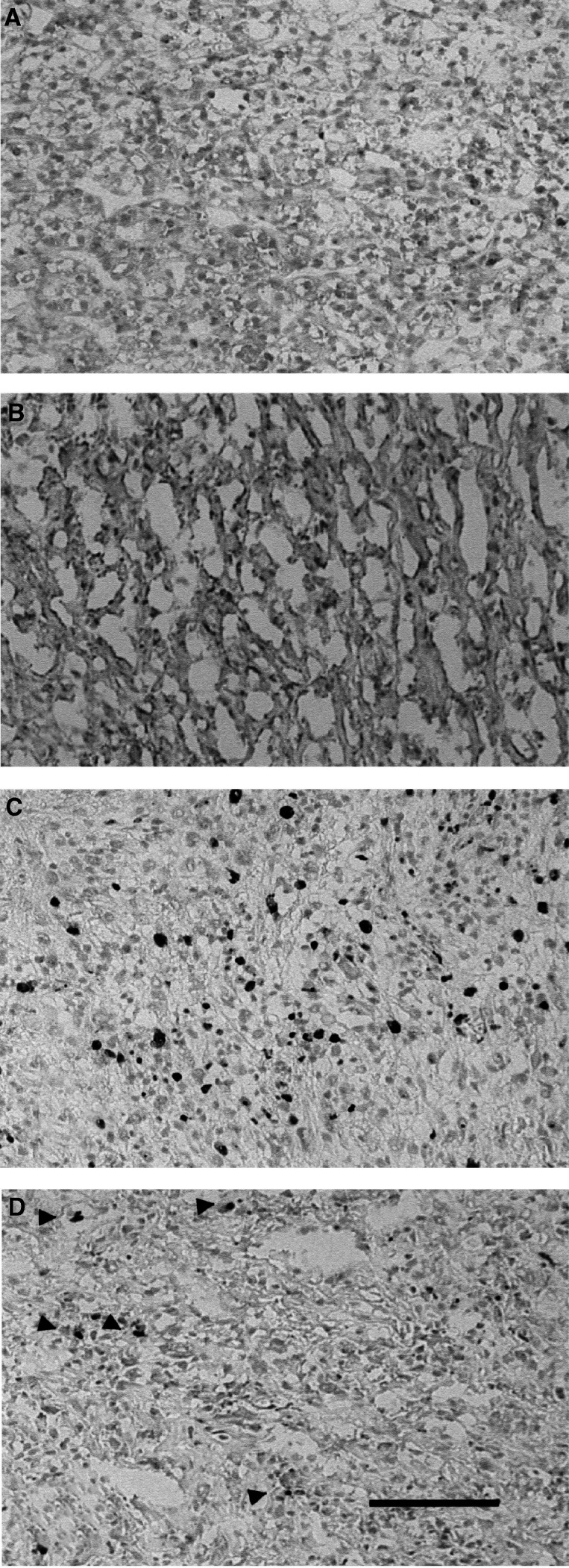
Immunohistochemical staining of (**A**) Bcl-2 (No. 101), (**B**) caspase-3 (No. 98), (**C**) Ki-67 (No. 98) of the indicated RCC tissue samples. (**D**) TUNEL staining of No. 98 sample; arrowheads point at some of TUNEL-positive nuclei. Scale bar is 100 *μ*m.

**Figure 2:**
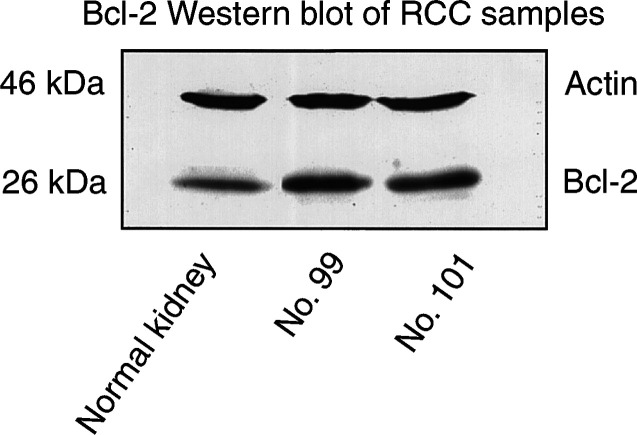
Western blotting analysis for Bcl-2 in normal kidney and RCC. Actin was applied as a control for loading.

Bcl-2 was expressed in the cytoplasm of cancer cells (Figure 1A

). The expression of Bcl-2 was detected in 72 of 101 (71.3%) cases. In contrast, the expression of p53 was detected in only 1 case in the nuclei and caspase-3 was detected in four cases in the cytoplasm of cancer cells (Figure 1B). Expression of Bcl-2 was confirmed by Western blot analysis (Figure 2

). The expression of Bcl-2 was detected more frequently in pT1-2 than pT3-4 (*P*=0.0301), and more frequently in G1-2 than G3 (*P*=0.0020, Table 3

**Table 3:** Correlation of Bcl-2 expression with stage or grade

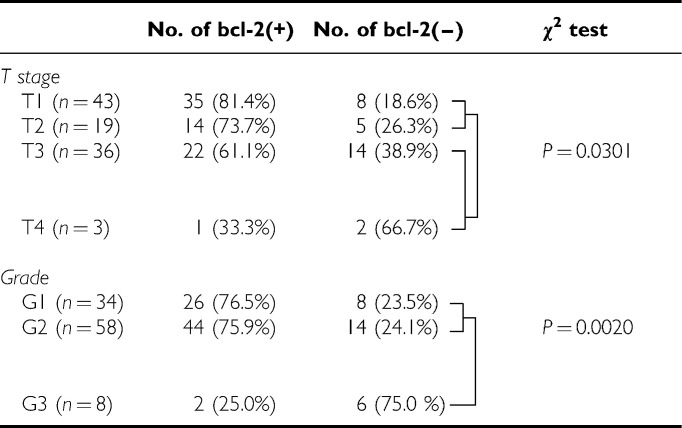

). Bcl-2-positive cases showed better prognosis (*P*=0.0014, Figure 3A

**Figure 3:**
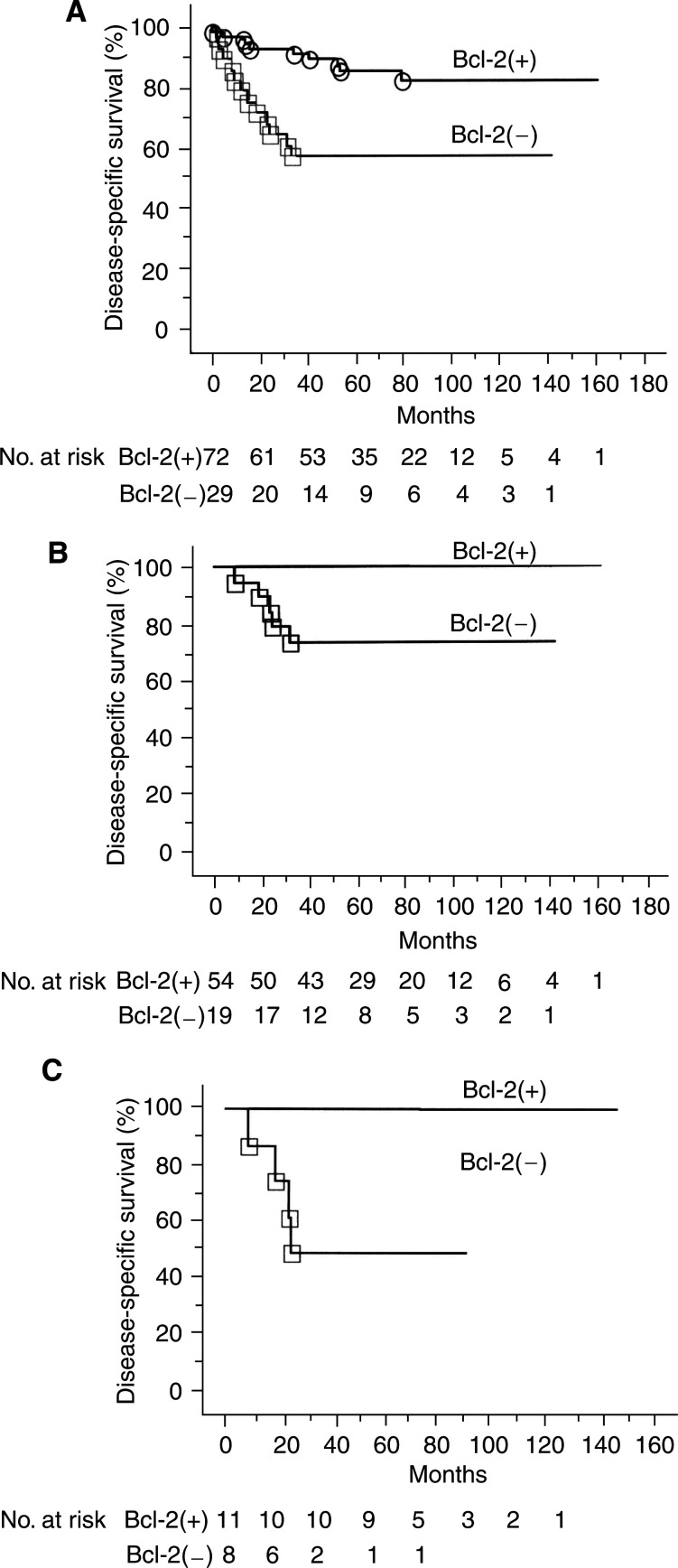
(**A**) Disease-specific survival of all cases according to Bcl-2 expression. (**B**) Disease-specific survival of curatively operated cases according to Bcl-2 expression. (**C**) Disease-specific survival of pT3-4 cases with curative operatation according to Bcl-2 expression.

). In 73 cases without metastasis at surgery, Bcl-2-positive cases also showed better prognosis and no Bcl-2-positive cases died of RCC (Figure 3B). When we compared each patient group based on T categories, in pT3-4 cases without metastasis at surgery, there was a more significant difference in survival between Bcl-2-positive and -negative cases (Figure 3C). In clear cell tumours which are the most common type of RCC, the expression of Bcl-2 was detected in 57 out of 76 (75.0%) cases and Bcl-2-positive cases also showed better prognosis (*P*=0.020).

### Detection of cell proliferation and apoptosis

Ki67 positivity was detected in the nuclei of cancer cells in all cases (Figure 1C). The PI ranged from 0.27 to 28.57% (mean 3.68%). The value of PI was significantly higher in G3 than G1-2 (*P*=0.0007) and T3-4 than T1-2 (*P*=0.0037). In 46 cases (45.5%), TUNEL-positive cancer cells were detected (Figure 1D). TUNEL-positive nontumour cells including lymphocytes were not counted. The AI ranged from 0 to 3.26% (mean 0.25%). As well as PI, the value of AI was significantly higher in G3 than G1-2 (*P*=0.0014) (Table 4

**Table 4:** Correlation of AI and PI with stage or grade

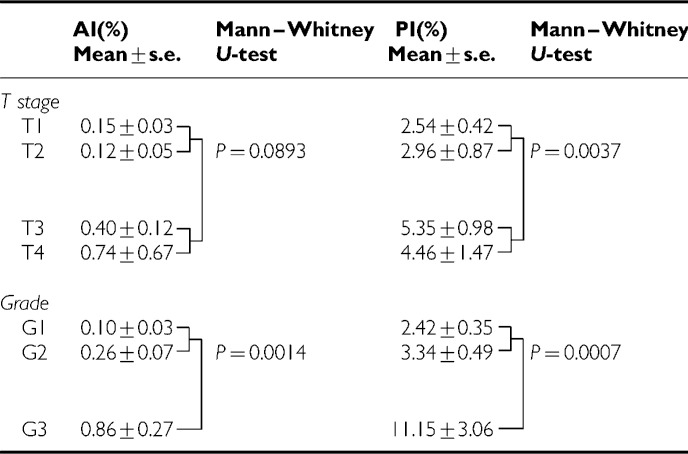

), and there was a positive relation between AI and PI (*R*=0.600, *P*<0.0001). Furthermore, both AI (*P*=0.0074) and PI (*P*<0.0001) were inversely correlated with Bcl-2 expression (Table 5

**Table 5:** Correlation of Bcl-2 expression with AI and PI

	**No. of bcl-2(+)**	**No. of bcl-2(−)**	**Mann-Whitney *U*-test**
AI (%) Mean±s.e.	0.135±0.028	0.546±0.146	*P*=0.0074
PI (%) Mean±s.e.	2.489±0.316	6.631±1.167	*P*<0.0001

).

### Multivariate analysis

Multivariate analysis was performed for the parameters which had a significant effect on prognosis using univariate analysis including Bcl-2 expression, AI, PI, stage and grade. Of the five parameters, PI (*P*=0.0012), stage (*P*=0.0004) and grade (*P*=0.0118) had significant effects on prognosis (Table 6

**Table 6:** Univariate and multivariate analysis of disease-specific survival in all cases

**Variable**	**Categories compared**	**Univariate *P*-value**	**Multivariate *P*-value**
Stage	T1-2 *vs*T3-4	<0.0001	0.0004
Grade	G1-2 *vs* G3	<0.0001	0.0118
Bcl-2 expression	Positive *vs* negative	0.0027	0.0879
AI	≦0.25 *vs* ≧0.25	0.0135	0.4738
PI	≦3.65 *vs* ≧3.65	<0.0001	0.0012

). In 73 cases without metastasis at surgery, only Bcl-2 expression had a significant effect on prognosis (*P*=0.0487, Table 7

**Table 7:** Univariate and multivariate analysis of overall survival in cases without metastasis at surgery

**Variable**	**Categories compared**	**Univariate *P*-value**	**Multivariate *P*-value**
Stage	T1-2 *vs*T3-4	0.0572	0.2750
Grade	G1-2 *vs* G3	0.0276	0.1176
Bcl-2 expression	Positive *vs* negative	0.0151	0.0487
AI	≦0.25 *vs* ≧0.25	0.0516	0.6474
PI	≦3.65 *vs* ≧3.65	0.0066	0.0732

) in multivariate analysis.

## DISCUSSION

Bcl-2 is a proto-oncogene known to be a negative regulator of apoptosis. It plays a prominent role in cell longevity through preventing apoptosis. High levels of Bcl-2 protein expression have been found in many different types of cancer, suggesting a possible role for Bcl-2 to deregulate apoptosis and promote malignant tissue transformation. Indeed, in urothelial transitional cell cancer (Bilim *et al*, 1998), oesophagus cancer (Hsia *et al*, 2001) and prostate cancer (Bauer *et al*, 1996) cases with Bcl-2 positivity represent poor prognosis. However, in RCC, several studies have shown that Bcl-2 positivity was not associated with prognosis (Lipponen *et al*, 1995; Vasavada *et al*, 1998; Huang *et al*, 1999; Sejima and Miyagawa, 1999; Zhang and Takenaka, 2000; Uchida *et al*, 2002). Unexpectedly, our present study showed that Bcl-2 positivity was associated with better prognosis. Moreover, multivariate analysis showed that, in cases without metastasis at surgery, Bcl-2 expression was an independent predictor of better prognosis on overall survival: in fact, in this subgroup, no Bcl-2-positive cases died due to RCC. With regard to correlation between Bcl-2 expression and pathological characteristics, Lipponen *et al* (1995) stated that Bcl-2 expression was related to low T category (Table 1). The present study also showed that Bcl-2 positivity was associated with low stage. However, Vasavada *et al* (1998) reported that high Bcl-2 expression showed a significant correlation with higher tumour grade (Table 1). Our findings were opposite to their observations. The reason is unknown; however, it may be explained by the number of cases or methods used including primary antibodies. They investigated a small subgroup including only 28 clear and seven papillary renal cell carcinoma cases (Table 1, Vasavada *et al*, 1998); on the other hand, we investigated a larger number of patients. To comfirm the specificity of the staining, Bcl-2 expression was analysed by Western blot. Indeed, in breast cancer (Kymionis *et al*, 2001; Sjostrom *et al*, 2002) and thyroid carcinoma (Viale *et al*, 1995), Bcl-2 positivity also showed a favourable outcome. These findings were unexpected based on *in vitro* data that comfirmed an antiapoptotic role for Bcl-2. Bcl-2 has been suggested to play an important role in tumorigenesis of RCC, because previous studies including our own reported that bcl-2 expression was seen in the majority of RCC; however, the results of the present study suggest an association of Bcl-2 expression with a less aggressive phenotype of RCC.

To gain further insight into the role of Bcl-2 in RCC and its relation with better prognosis, we analysed AI, PI and the expression of caspase-3, p53 and correlations between Bcl-2 expression and these parameters. Bcl-2 expression was significantly associated with low AI. Furthermore, three of four cases with caspase-3 expression were Bcl-2 negative with high AI. Although only four of 101 specimens were caspase-3 positive, this might suggest caspase-3 involvement in apoptosis of tumour cells without Bcl-2 expression. Regarding the correlation between Bcl-2 expression and tumour-proliferative activity, an inverse correlation between them was reported in breast (Bozzetti *et al*, 1999), lung (Ishida *et al*, 1997) and endometrial carcinomas (Kuwashima *et al*, 1997). In the present study, Bcl-2 expression was also associated with low PI, which implies that Bcl-2 might be a negative controller of proliferation in RCC. This hypothesis was supported by previous findings that showed that Bcl-2 inhibited cell proliferation using bcl-2 trangenic mice (Pierce *et al*, 2002). Moreover, in the present study, there was a significant positive relation between AI and PI, and the result was consistent with previous findings (Tannapfel *et al*, 1997; Zhang and Takenaka, 2000).

The following model is suggested to the present findings. RCC with a high rate of cell proliferation progresses rapidly to high stage and grade and results in the patient's death due to the disease. At the same time, such RCC demonstrates a high rate of spontaneous apoptosis due to accumulation of genetic alterations, hypoxia inside the tumour and other factors. Bcl-2 prevents both cell proliferation (Pierce *et al*, 2002) and apoptosis. Thus, Bcl-2-positive tumours may be slow growing and so possess less malignant potential. On the other hand, Bcl-2 as an antiapoptotic molecule protects tumour cells from induced apoptosis and may render RCC resistant to all kinds of apoptotic–triggering therapeutics, including chemotherapy and irradiation.

In conclusion, Bcl-2 expression may be a novel prognostic factor for better outcome of RCC patients. In cases without metastasis at surgery, Bcl-2 expression is an independent predictor of better prognosis.
